# The Role of Long Non-Coding RNA in Anxiety Disorders: A Literature Review

**DOI:** 10.3390/ijms26115042

**Published:** 2025-05-23

**Authors:** Laura Dayanara López-Rocha, Armando Ruiz-Hernández, Gustavo Martínez-Coronilla, Ana Gabriela Leija-Montoya, Mario Peña-Peña, Fausto Sánchez-Muñoz, Ulises Rieke-Campoy, Javier González-Ramírez

**Affiliations:** 1 Facultad de Medicina Mexicali, Universidad Autónoma de Baja California, Centro Cívico, Mexicali 21000, BC, Mexico; dayanara.lopez@uabc.edu.mx (L.D.L.-R.); armando.ruiz.hernandez@uabc.edu.mx (A.R.-H.); gustavoj@uabc.edu.mx (G.M.-C.); gabriela.leija@uabc.edu.mx (A.G.L.-M.); 2 Laboratorio de Biología Molecular, Centro de Ciencias de la Salud, Unidad Universitaria, Calle de la Claridad, S/N, Col. Plutarco Elías Calles, Mexicali 21376, BC, Mexico; 3 Departamento de Fisiología, Instituto Nacional de Cardiología, Juan Badiano No. 1, Col. Sección XVI, Tlalpan 140080, DF, Mexico; marionutricion2017@gmail.com (M.P.-P.); fausto22@yahoo.com (F.S.-M.); 4 Facultad de Enfermería, Universidad Autónoma de Baja California, Av. Álvaro Obregón y Calle “G”, S/N, Col. Nueva, Mexicali 21100, BC, Mexico; rieke@uabc.edu.mx

**Keywords:** anxiety disorders, non-coding RNA, long non-coding RNA, neuroinflammation

## Abstract

Anxiety is a fear response that triggers a stress reaction with the purpose of defending against or avoiding danger, which is considered physiological, until it becomes excessive and persistent, affecting daily life activities. Non-coding RNAs have been explored in terms of their relationship with diseases, and several of them, such as miRNAs, have been found to be key factors in the development of diseases. Specifically, the expression of long non-coding RNAs (lncRNAs) has been implicated in the development of anxiety through various mechanisms such as nervous system development, synaptic function, neurotransmitter regulation, and neuroinflammation. However, several recent reviews have explored the roles of lncRNAs in various mental diseases (mainly in schizophrenia), and considering that existing reviews do not cover the interaction between lncRNAs and aspects such as neuroimmunity in anxiety disorder pathophysiology, the aim of this literature review is to summarize the current knowledge about the contributions of lncRNAs to the molecular and cellular mechanisms underlying the pathogenesis of anxiety disorders. Additionally, we explore their potential applications in the diagnosis, as well as possible treatment approaches, of these disorders, challenges, and current limitations.

## 1. Introduction

Anxiety disorders (ADs) are amongst the most common mental disorders worldwide, affecting 374 million people; the overall prevalence varies significantly between countries due to different methodological approaches, ranging from 3.8 to 25%, it should also be noted that after the COVID-19 pandemic, rates of ADs increased by 25% worldwide [1,2]. Regarding their prevalence by sex, it is estimated to be higher in women (30.5%) compared to men (19.2%) [3]. It is known that they generally begin before or in early adulthood, typically between the ages of 10 and 24; as for its global trend, we know that there has been an important increase of 52% in ADs from 1990 to 2021, with a peak among the 20 to 24 age group [4,5]. According to the Global Burden of Diseases, ADs are the sixth leading cause of disability-adjusted life years in the 10 to 24 age group [6].

The definition tells us that anxiety is a fear response that triggers a fight-or-flight behavior with the purpose of defending or avoiding danger, which is considered physiological [7]. Nevertheless, anxiety becomes pathological when this response becomes excessive and persistent, also accompanied by symptoms such as tachycardia, palpitations, nausea, among others [2,8].

The complexity of anxiety has led to it being divided into different anxiety disorders, and each disorder presents distinct clinical characteristics that aid in its diagnosis and guide the selection of the most appropriate treatment approach [2,9].

Regarding their pathological origins, ADs are multifactorial in nature; although many aspects remain to be elucidated, their estimated heritability ranges between 20 and 50%, depending on the subtype and population studied [5,10,11,12]. Studies have shown that psychosocial and environmental factors play a major role in the development of these disorders [3,11,13].

In fact, it is currently known that the presence of environmental factors such as prenatal stress, early-life adversity, trauma, diet and toxin exposure, contribute significantly to individual susceptibility to anxiety disorders [14,15,16]. These environmental insults can induce epigenetic modifications, which have long-lasting effects on neurobiological circuits associated with pathological anxiety [17].

Epigenetics is currently recognized as a crucial factor in the development of anxiety, as, by its definition, it is known as the study of changes in gene expression that do not alter the DNA sequence, and it is being studied to determine its contribution to AD pathogenesis [14]. Several epigenetic mechanisms are known to be involved, such as histone modification, chromatin remodeling, and non-coding RNA (ncRNA) expression [3].

Among the epigenetic mechanisms, one that has taken on great importance is non-coding RNAs (ncRNAs), the study of which has moved from merely identifying a few specific molecules to a broader exploration of their functions in various biological processes [18].

Non-coding RNAs (ncRNAs) have been defined as transcripts from genes that are not translated into proteins; these single-stranded ribonucleic acid sequences are normally divided according to their size [19].

However, this classification by size is currently considered to present several problems, especially if based on a threshold such as 200 nt, since their size is not a definitive limit, because some RNAs within this range may have coding functions [20]. Another problem is that lncRNAs can have diverse lengths, so considering only size could overlook highly important functional characteristics or nuances in their interactions [20].

That is why, starting in 2023, several authors have begun to recommend using another classification to divide them into small ncRNAs (<50 nucleotides (nt)), RNA polymerase III transcripts (50–500 nt), and long ncRNAs (>500 nt), mostly generated by RNA polymerase II [20]. It is worth mentioning that the previous cut-off size of more than 200 nt for lncRNA is still being used.

In this way, in this review we will use the definition of long non-coding RNAs (lncRNAs) being transcripts longer than 200 nt [20]. Their biological importance lies in the fact that their expression patterns are highly dynamic during cell differentiation and development, playing a key role in cellular function and maintaining tissue homeostasis. Similarly, abnormal lncRNA expression has been linked to the pathogenesis of various disorders [21,22].

In addition to the above, it should be noted that lncRNAs are highly heterogeneous in their origin since they can be (1) intronic, encoded in gene introns; (2) intergenic, encoded between two genes; (3) enhancers, encoded in gene promoters; (4) bidirectional, encoded nearby to a gene of the opposite strand; (5) sense-overlapping, encoded in the exons and introns of different genes in the sense strand; and (6) antisense, which are encoded in the antisense strand [23]. They can also be derived from the mitochondrial genome [24].

As for their specific functions, these are diverse since lncRNAs can act at both transcriptional and post-transcriptional levels, being able to act in epigenetic regulation, chromatin remodeling, and protein metabolism control [21]. It should be noted that they regulate gene expression in a highly versatile manner due to their ability to establish physical and functional interactions with DNA, RNA, and proteins [21,25]. LncRNAs are signaling molecules that integrate stimulus-specific cellular information, enabling and regulating the temporal, spatial, and developmental coordination of various cellular functions [24].

Since we consider that existing reviews do not cover the interaction between lncRNAs and neuroimmunity in anxiety disorder pathophysiology [16,17,26,27,28], the aim of this literature review is to summarize current knowledge about the contributions of lncRNAs to the molecular and cellular mechanisms underlying the pathogenesis of anxiety disorders. Additionally, we explore their potential applications in the diagnosis, and possible treatment approaches, of these disorders, current challenges, and limitations.

## 2. Literature Screening Methods

A literature search was performed between January and April of 2025. No time restrictions were imposed as a search criterion. The PUBMED, SCOPUS, Google Scholar, MDPI, ResearchGate, and Web of Science databases were utilized, using a combination of search terms: anxiety disorders; non-coding RNA; long non-coding RNA; neuroinflammation; and neurodevelopment. Inclusion criteria were the use of the English and Spanish languages and publications concerning lncRNAs in human anxiety, as well as in vitro or animal models mimicking the characteristics of anxiety. Emphasis was placed on the identification of lncRNAs with validated expression by quantitative real-time polymerase chain reaction (qRT-PCR), biological processes modulated by these ncRNAs, and experimental analyses of the involved molecular mechanisms and molecular targets.

## 3. Long Non-Coding RNA in the Pathogenesis of Anxiety Disorders

### 3.1. Anxiety Pathogenesis

ADs have multiple factors that influence their development, among which we find genetic factors, as some genome-wide association studies have pointed out some single-nucleotide polymorphisms (SNPs) on genes, such as STAB1, ESR1, and MAD1L1, among others [29].

Another important factor for developing anxiety are environmental factors, such as parenting styles, traumatic experiences, social pressures, negative life events, work stress, and aversive social interactions, which can contribute to the development of anxiety disorders [30].

It is currently known that the interactions between these factors can lead to epigenetic changes through transcriptional and post-transcriptional mechanisms; these changes entail altered brain function by modifying expression patterns, leading to altered regulation of the neuroendocrine system, such as the functioning of the hypothalamus–pituitary–adrenal (HPA) axis (crucial for stress responses) [31].

Synaptic plasticity is a neuronal process that involves synaptic density modifications to ensure neuronal circuit stability and adaptability in response to environmental stimuli; it is essential for memory, learning, and resilience to stressful and demanding situations. When this process is altered, the development of anxiety is promoted [17,32,33].

Another very important effect is the presence of neuroinflammation. This inflammation, which can be caused by chronic stress or other factors, can affect the brain regions involved in emotional regulation and fear responses, leading to increased anxiety and anxiety-like behaviors [34].

In fact, it is known that chronic stress and persistent hypercortisolemia can contribute to neuroimmune dysregulation, characterized by the overactivation of the inflammatory response, and since inflammation and anxiety are interconnected, they both exacerbate each other [28,35]. In effect, what happens is that stress triggers inflammation, and the inflammatory markers produced by this inflammation can influence the brain regions involved in mood and anxiety. As a result, anxiety worsens inflammation, which further intensifies anxiety symptoms [36].

Other factors are known to contribute to anxiety, such as neuropeptides, which play an important role in neural pathways and the neuroendocrine system response to stress regulation [37]. Oxytocin, composed of nine amino acids, is released in response to acute stress, leading to the activation of GABAergic interneurons in the amygdala; it also reduces the expression of corticotropin-releasing hormone (CRH) mRNA, thus lowering the neuroendocrine stress response, but the anxiolytic effect may vary among males and females [37,38]. In the medial prefrontal cortex, oxytocinergic interneurons produce CRH binding protein (CRHBP) and GABA; in male rodents, stimulation of these interneurons induces the release of CRHBP, leading to stress reduction, whereas in female rodents, which have higher levels of CRH, the release of CRHBP was not able to effectively inhibit its activity [39].

**Figure 1:**
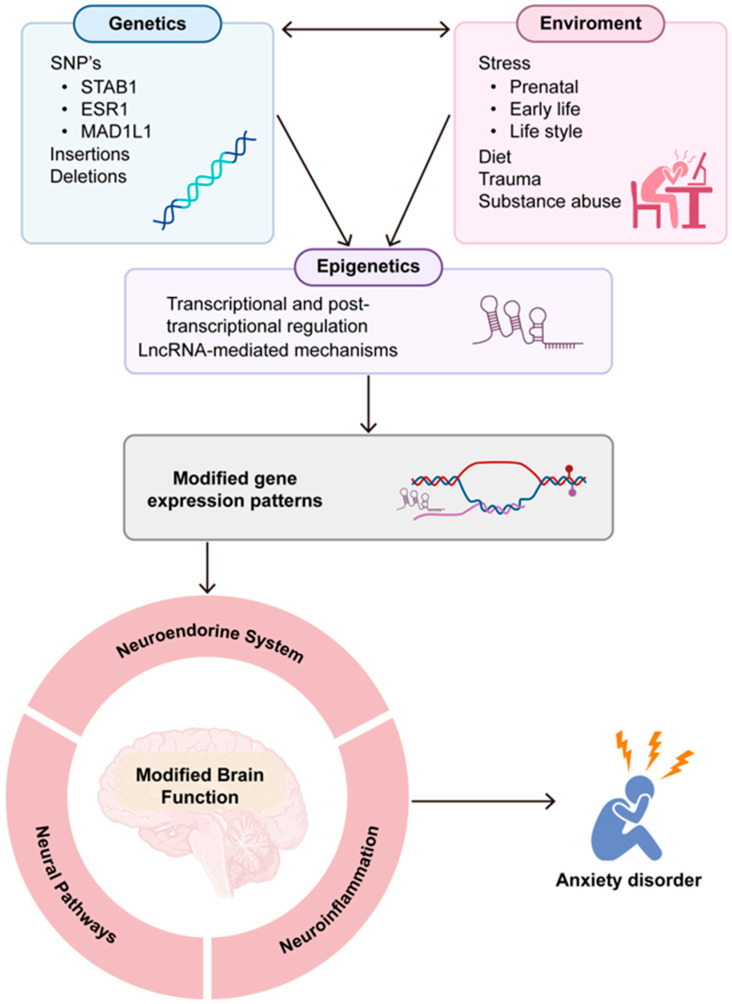
Risk factors related to anxiety disorder development. ADs have multiple factors that influence their development. Genetic factors have been shown to increase someone’s susceptibility to developing ADs; some genome-wide association studies have pointed to single-nucleotide polymorphisms (SNPs) on genes, such as STAB1, ESR1, and MAD1L1 among others, but studies on twins have shown that environmental factors play a major role in their development. Genetic and environmental factors can lead to epigenetic modifications through to transcriptional and post-transcriptional regulation mechanisms, which can be mediated by long non-coding RNAs. Genetic, environmental, and epigenetic factors can result in altered brain function by modifying brain expression patterns, leading to altered regulation of the neuroendocrine system, such as functioning of the hypothalamus–pituitary–adrenal (HPA) axis, crucial for stress responses, neural pathways, including neurotransmitter release, neural plasticity, and regulation of neural stress, and neuroinflammation, by activation of the microglia and other glial cells, cytokine release, and neuron apoptosis. Neural plasticity modifications and neuron apoptosis may also lead to changes in the brain structure. The neuroendocrine system, neural pathways, and neuroinflammation have a very complex interaction between them, and alterations in one of them may induce changes in the other two through epigenetic regulation mechanisms. The addition of these changes can lead to AD development. Created in BioRender: accessed on 21 April 2025. https://BioRender.com/vbjh0g8.

Many of the disorders described above are affected by the dysregulation of non-coding RNAs, for example, microRNAs (miRNAs) and long non-coding RNAs (lncRNAs) [40]. Due to the above, in the following paragraphs we will try to describe the contributions of lncRNAs to these factors that participate in the pathogenesis of anxiety. Figure 1 is a schematic representation of the diverse factors related to anxiety disorder development.

### 3.2. Participation of lncRNAs in the Regulation of Neurodevelopment 

Neurodevelopment is a dynamic and complex process requiring precise stem cell proliferation and differentiation, which is coordinated by factors such as genetic, biochemical, physical, and environmental factors, from early embryonic stages to postnatal life [41,42]. LncRNAs are closely related to this process and help regulate brain function as well as formation [43]. This has been analyzed in some works, for example, a study conducted by Zou et al. on a Sprague Dawley rat model reported that, by putting them on a high-fructose diet during gestation and lactation, lncRNA expression can be altered in diverse ways, measured via massive sequencing in the hippocampus of the offspring, and these changes in expression could subsequently affect anxiety-related behavior [16].

On the other hand, it has been observed that the brain-derived neurotrophic factor antisense RNA (BDNF-AS) controls neurogenesis, since the overexpression of the BDNF gene is associated with different stages of neuronal development in both in vitro and in vivo models [41,42]. In this sense, BDNF-AS is an important regulator of neurogenesis and neuroplasticity, both important in mitigating anxiety behaviors [43,44].

Another lncRNA important for neurogenesis is Embryonic Ventral Forebrain 2 (Evf2), which was the first lncRNA specific to the central nervous system (CNS) to be characterized in vivo [42]. This lncRNA helps with the differentiation of GABAergic neurons, since it eliminated the gene that encodes this lncRNA in a mouse model; mice with the deletion showed an imbalanced excitatory activity in their postnatal hippocampus and dentate gyrus [45].

Another lncRNA related to neurogenesis is PNKY [42]. This lncRNA was described by Ramos in 2015 and it was shown that it was in the nucleus of dividing neural stem cells (NSCs) in mouse and human brains [46]. It seems that its function depends on controlling and balancing neuronal self-renewal and differentiation in embryonic NSCs, mostly via alternative splicing pathways [42,46].

Regarding neuroplasticity, an involved lncRNA is BC1/200, which is highly expressed in neuron dendrites and modifies synapse composition as a reaction to neuronal activity, playing an important role in long-term changes due to neuroplasticity modulation; the absence of this lncRNA in mice has been associated with neuronal hyperactivity and anxiety [42].

The lncRNA metastasis-associated lung adenocarcinoma transcript 1 (MALAT1) is abundantly expressed in neurons; in particular, its role in neuroplasticity has been explored, and there are studies that mention that it can modulate this. In fact, when the MALAT1 gene is eliminated in mice, the result is a decrease in synaptic density [47,48,49].

Another lncRNA that is expressed in the brain cortex, the nuclear paraspeckle assembly transcript 1 (NEAT1) lncRNA, is emerging as a sensor of stress signals and may play a role in neuroplasticity, for example, the overexpression of this lncRNA in rodents induced impaired memory formation, which is an important process for developing adaptative behaviors to stress [50,51,52].

The lncRNA GOMAFU, also known as myocardial infarction-associated transcript (MIAT), is highly expressed in differentiating neural progenitor cells, retinal cells, cerebral cortex pyramidal cells, and the hippocampus, and can participate in several neurodevelopmental processes such as neural and glial cell differentiation, the survival of newborn neurons during cortex formation, and cognitive decline in aging [53,54]. All the processes and studies already mentioned are summarized in Table 1.

The specific temporal and spatial expression of lncRNAs, along with their capacity to interact with proteins, DNA, and other RNA molecules, makes them key elements in achieving proper nervous system formation, organization, and function [24,32,58]. Although the relationship between neurodevelopment and anxiety is complex and involves several genetic, epigenetic, and environmental factors, evidence shows that lncRNAs are implicated in many critical steps of brain development and function. Consequently, their impaired expression may lead to the development of psychiatric disorders, such as ADs.

### 3.3. Influence of lncRNAs on Neurotransmission 

LncRNAs can modulate neuronal excitability and brain circuit function in ways that influence anxiety-related behaviors [59]. The precise regulation has not been fully elucidated, but it appears to be crucial for maintaining normal central nervous system (CNS) function and modulating behavioral responses, including anxiety [24].

LncRNAs help regulate the expression of genes involved in synapse formation and function [32]. They influence synaptic density and transmission strength, and can also interact with chromatin remodeling complexes, directing them to specific genomic regions to activate or repress these genes [24]. Given that synaptic activity is tightly linked with neuronal survival and brain structure, the dysregulation of these processes may contribute to ADs [35,60]. Notably, one of the most significant risk factors for developing anxiety disorders is exposure to stress, which has been associated with impaired synaptic activity, neuronal apoptosis, and structural remodeling in brain regions such as the hippocampus [60,61,62].

The antisense lncRNA Evf2 promotes the expression of DLX5 and DLX6, transcription factors required for the development of GABAergic neurons and interneurons [63]. The dysregulation of Evf2 is associated with a decrease in GABA interneurons, which induces important inhibitory signaling to help counterbalance excitatory activity to maintain neural stability [60,64]. Amygdala neural hyperactivity has been associated with anxiety-like behaviors and ADs [28].

The lncRNA GOMAFU is involved in dopamine receptor 2 and glutamate receptor 3 pathways; evidence has shown that the downregulation of this lncRNA enhances the expression levels of both pathways, altering brain excitability, and has been suggested as a brain modulator of anxiety behaviors [59,64]. In a mouse model, the silencing of the lncRNA GOMAFU by antisense oligonucleotides (ASOs) in the prefrontal cortex was associated with anxiety-like behaviors [65]. It also plays a role in the regulation of alternative splicing through interactions with splicing factors and negatively regulates genes that translate to proteins that promote sustaining anxiety [21,59].

The lncRNA NEAT1 regulates paraspeckle structures and is involved in modulating neural excitability [32,66]. In a study by Kukharsky et al., conducted on a mouse model, they found that NEAT1 downregulation induces neural hyperactivation, especially in cortical regions, and is associated with panic responses [51].

BDNF-AS is an antisense lncRNA that acts as a negative regulator of BDNF by suppressing its mRNA expression [67]. Elevated BDNF-AS levels promote neurotoxicity, increase apoptosis, and decrease cell viability [68]. BDNF itself is dynamically regulated by stimuli such as excitatory synaptic activity, hormones, and neuropeptides [44,69]. It plays a key role in the proper GABAergic and glutamatergic systems and influences dopaminergic as well as serotoninergic neurotransmission [44].

The mitochondrial lncRNA 7S is known as a gene regulator capable of controlling mitochondrial transcription [70]. A decrease in 7S RNA levels is associated with reduced mitochondrial translation and mitochondrial dysfunction; however, the precise mechanism remains unknown [71]. In a study conducted by Wang et al., the higher expression of 7S RNA was observed in patients with clinical anxiety and depression compared to healthy controls, with a significant reduction following treatment [72].

LncRNAs influence neuromodulation and brain signaling by regulating gene expression, participating in synaptic plasticity, interacting with splicing proteins and contributing to intercellular communication [59]. The dysregulation of brain signaling pathways is linked to AD pathogenesis, but neuroinflammation and lncRNAs also appear to be common ground [40,59].

### 3.4. Neuroinflammation Related to Anxiety

lncRNAs exert a significant influence on neuroinflammation through diverse mechanisms such as gene expression regulation, microglial activation as well as modulation, involvement in key signaling pathways, and blood–brain barrier permeability control [28,34,40,73]. Their dynamic and brain-specific expression underscores their critical role in neuronal function and the pathogenesis of various neuropsychiatric disorders, including ADs [21].

Neuroinflammation exacerbates HPA axis dysfunction and promotes structural as well as functional alterations in key brain regions involved in anxiety, particularly the hippocampus and amygdala [33]. The hippocampus, a structure sensitive to chronic stress, undergoes neuronal apoptosis and reduces neurogenesis, shifting stress processing toward the amygdala and reinforcing hyperactive fear responses [60,61,62].

Cytokines are signaling molecules that immune cells release to regulate inflammation [74]. Pro-inflammatory cytokines (IL-1β, IL-6, and TNF-α, for example) promote and sustain inflammatory processes; on the other hand, anti-inflammatory cytokines (like IL-4, IL-10, and TGF-β) help in the resolution of inflammatory processes, and the balance between these two kinds of cytokines determines the immune response [34,74,75]. Immune cells communicate by means of autocrine and paracrine signaling; the microglial response differs between neurotransmitters, local tissue environments, and receptor types [74].

Studies have shown that chronic stress can lead to neuroinflammation; in the hippocampus, the pro-inflammatory state activates NLR family pyrin domain containing 3 (NLRP3) inflammatory vesicles, after which NRLP3 assembles caspase-1 activation complexes, also known as NLRP3 inflammasomes, to cut the precursor of interleukin 1B (IL-1B) and release the active form of this cytokine [76,77]. In mice exposed to acute stress, hippocampal microglia tend to release higher levels of tumor necrosis factor alpha (TNF-α) and IL-1B, which inhibits neurogenesis and promotes neural apoptosis, thus enhancing anxiety behaviors [77,78,79,80]. In a meta-analysis of inflammatory mediators in patients with generalized anxiety disorder, a higher number of immune cells producing pro-inflammatory cytokines, such as TNF-α, IL-2, and interferon-gamma (IFN-γ), was found [74].

Furthermore, inflammation has been shown to directly enhance amygdala activity, predisposing individuals to heightened responses to stressors [33,81,82]. In a meta-analysis by Etkin and Wager concerning functional magnetic resonance imaging and positron emission tomography, patients with anxiety disorders, such as post-traumatic stress disorder (PTSD), atopic phobia, and social anxiety disorder, demonstrated stronger activity in the amygdala and insula compared to healthy controls [83].

Research has demonstrated a strong connection between amygdala activity, inflammation, and anxiety-related behaviors [81,84]. Acute social stress, such as during an interview, has been linked to increased amygdala activity and heightened connectivity with the dorsolateral prefrontal cortex, which correlates with elevated levels of pro-inflammatory cytokines like IL-6 and TNF-α [84]. Supporting this, Zheng et al. found that microglial activation and the subsequent production of pro-inflammatory cytokines in the amygdala contributed to an excitatory/inhibitory imbalance in a mouse model of lipopolysaccharide-induced neuroinflammation, accompanied by increased presynaptic glutamate release, resulting in anxiety- and depression-like behaviors [28].

Notably, increased amygdala activity can further amplify inflammation, creating a positive feedback loop that exacerbates the imbalance between excitatory glutamatergic transmission and inhibitory GABAergic pathways [26]. This sustained dysregulation promotes prolonged microglial activation and the release of additional pro-inflammatory cytokines, perpetuating neuroinflammation [34].

The anti-inflammatory cytokine IL-10 has been shown to reverse abnormal GABA transmission in the amygdala, mitigating anxiety-like behaviors and substance dependence [85]. Additionally, in adult male Sprague Dawley rats, repeated social defeat stress triggered microglial activation in the amygdala, leading to increased discharge in this region. Remarkably, blocking microglial activation prevented anxiety-like behaviors in these rats [26,85]. Reduced levels of IL-10 have been described in patients with symptoms of anxiety, depression, and suicidal risk in a population study [75]. 

In a study conducted on a mouse model, the results showed the overexpression of IL-4 in the mice group with low stress susceptibility [75]. Microglia exposed to IL-4 and IL-13 specializes to the M2 subtype, known for its anti-inflammatory functions [75].

Neuroinflammation in the CNS is primarily driven by microglia, astrocytes, and endothelial cells [86]. Increasing evidence highlights the significant role of lncRNAs and other ncRNAs in modulating this inflammatory response, as they can exert pro- or anti-inflammatory effects by regulating key signaling pathways such as NF-κB, PI3K/AKT, JAK/STAT, MKK4-JNK, and TLR [87,88,89]. A summary of lncRNA mechanisms associated with neuroinflammation is shown in Table 2.

The NF-κB pathway plays a central role in controlling neuroinflammation via modulating the expression of chemokines, cytokines, and other pro-inflammatory mediators [98]. Several lncRNAs become dysregulated under pathological conditions in the CNS, influencing NF-κB activity to either promote or inhibit neuroinflammation. Many of these lncRNAs can employ multiple mechanisms to modulate NF-κB signaling in a context-dependent manner. Examples of such lncRNAs include MALAT1, Gm4419, LincRNA GAS5, TUG1, and RMST, all of which regulate neuroinflammation through the NF-κB pathway [88].

IFN-γ contributes to stress-induced immune dysregulation [75]. In a group of women with breast cancer, after breast surgery and before adjuvant therapy, the ones with higher subjective stress showed lower basal and IFN-γ-induced NK cells in addition to reduced T cell proliferative responses to mitogens [75]. The nuclear lncRNA GOMAFU has been shown to act as a suppressor of the IFN-γ pathway. Its dysregulation appears to promote neuroinflammation and has been associated with anxiety-like behaviors in mouse models [55].

In a study conducted by Cheng et al., they found that neuregulin receptor-degrading protein 1 can mediate the ubiquitination of myeloid differentiation factor 88 (MYD88), thereby downregulating microglial activation and the subsequent release of inflammatory factors [96]. This process is regulated by the lncRNA HOTAIR, which was found to be highly expressed in activated microglia [96].

Chronic-stress-induced neuroinflammation appears to exacerbate dysfunction in key brain regions like the hippocampus and amygdala, contributing to anxiety-related behaviors [34,60]. The interplay between amygdala hyperactivity and inflammation creates a self-perpetuating cycle that sustains anxiety behaviors, underscoring the potential of lncRNAs as therapeutic targets for stress-related and inflammatory brain disorders.

## 4. Long Non-Coding RNA as a Potential Diagnostic Biomarker

Currently there are no standardized studies that demonstrate biomarkers that might permit the diagnosis of anxiety. Consequently, diagnosis is clinical and depends on criteria and scales with which to characterize symptoms [99].

A biomarker is a well-defined characteristic that is measured as an indicator of normal biological processes, pathological processes, or as a response to an intervention or exposure [100]. A diagnostic biomarker can detect or confirm the presence of a disease, condition, or a subtype of a certain disease [101]. Other classes of biomarkers are monitoring, pharmacodynamic/response, prognostic, safety, and susceptibility/risk biomarkers [102]. They must have high specificity and sensitivity for a particular condition, quantifiable with ease in approachable body fluids (non-invasive), for example blood, urine, or saliva, and stable in vivo as well as in vitro [102].

LncRNAs have potential use as biomarkers in the diagnosis, prognosis, and monitoring of diverse diseases, such as cancer, psychiatric disorders, metabolic disorders, cardiac diseases, and infectious diseases, among others [23]. Many lncRNAs are differentially expressed in a tissue-specific manner, and several lncRNAs are brain-expressed, suggesting that they are ideal candidates to serve as biomarkers of psychiatric disorders [103]. They are also relatively stable molecules, which may facilitate their detection and quantification in clinical samples; therefore, lncRNAs can be detected in body fluids like blood, allowing for their use as non-invasive [103].

In fact, there has been a continuous search for lncRNAs to use as biomarkers in different diseases; however, they are not yet standardized as they keep being researched, some examples of which are summarized in Table 3.

Something that would be very important to determine for a biomarker of brain diseases is to see if the changes produced in the brain can be reflected in the blood, since it is considered that blood allows us to obtain a sample that helps us to monitor the progression of diseases [112].

Therefore, it is important to mention that although it has not yet been fully established that there is a difference between the expression of lncRNAs detected in blood and their expression in the brain, some studies have shown that this difference exists and that, therefore, although blood may not accurately reflect lncRNA expression in the brain, some can be identified in the blood and potentially used as biomarkers of changes in the brain [113]. For example, there are studies on ischemic stroke where it was found that hypoxia caused alterations in the profiles of lncRNAs in the brain, but also in those found in circulation [114].

This has also been observed in other diseases, such as Alzheimer’s, where the upregulation of a lncRNA that has been implicated in the regulation of beta-secretase 1 (BACE1), an enzyme involved in the formation of amyloid plaques, a common pathobiochemical event underlying several debilitating human diseases, including Alzheimer’s, has been found [115,116].

In this way, with what has been reviewed, we can say that, while there is increasing evidence of lncRNA expression patterns in ADs and although results from studies carried out on cell and animal models have been encouraging, they have not yet been validated with human models, and their sensitivity as a potential biomarker is therefore unclear [48,55,65,91].

This limited understanding reflects the broader challenges in lncRNA research. Unlike sRNAs and miRNAs, lncRNAs are harder to characterize because of their larger size, higher structural complexity, and versatile action mechanisms [17,32,63,88]. In contrast, sRNAs and miRNAs have been studied more extensively due to their small sizes, simpler mechanisms, and earlier evolutionary origins [24,32]. In Table 4, studies on altered lncRNA and miRNA expression patterns in various anxiety-related disorders are summarized.

However, the constant improvement of lncRNA research holds promise for elucidating its roles in inflammatory processes and pathogenesis, particularly in uncharacterized conditions where their involvement remains poorly understood, such as ADs.

## 5. Targeting Long Non-Coding RNA

Given their importance in gene regulation and the pathogenesis of diverse diseases, lncRNAs have become promising therapeutic targets [121]. Several strategies have been developed as potential modulators of lncRNA function.

### 5.1. Antisense Oligonucleotides (ASOs)

ASOs specifically aimed to target and therefore inhibit lncRNA function or reduce its levels. This approach has shown efficacy in modulating the expression of genes and could have therapeutic implications in psychiatric and neurodegenerative disorders [22].

### 5.2. Small Interfering RNAs (siRNAs)

SiRNAs can selectively silence lncRNAs via the RNA interference pathway. Small-molecule drugs may also be promising therapeutic options for the treatment of certain diseases by specifically targeting lncRNAs and disrupting their interactions with either proteins or molecules [22].

### 5.3. Natural Antisense Transcripts (NATs)

Another promising approach is the in vivo inhibition of NATs, associated with a concerted increase in gene transcription for some genes [122]. LncRNA can be used to restore the function of other lncRNAs that are lost or downregulated in disease conditions, by mimicking their activity. Interestingly, the in vivo inhibition of NAT lncRNAs further activates gene transcription, presenting additional therapeutic opportunities [122].

### 5.4. MiRNAs and LncRNAs

For instance, MALAT1 can be degraded in the nucleus after targeting miRNA-9, which shows that miRNA manipulation could potentially have an indirect impact on the function of lncRNAs [123]. The use of lncRNAs as “molecular sponges” may directly influence critical signaling cascades and gene expression profiles, and so the targeting of lncRNAs could modulate these pathways [124].

### 5.5. Other Molecular Approaches

New delivery approaches that utilize exosome-based systems may provide a solution for lncRNA-targeting therapies, especially for diseases within the central nervous system [125]. Moreover, CRISPR-Cas9 technology can be used for the gene editing of lncRNA gene sequences, leading to their inactivation or modification [126].

The therapeutic targeting of lncRNAs is promising, but the field is still in its early stages. Further research is crucial to fully elucidate the complex functions and mechanisms of lncRNAs before these approaches can be translated into clinical practice.

## 6. Discussion

After reviewing the literature, we can say that there is still a long way to go to connect the expression of lncRNAs and ADs, although we must mention that, according to our analysis, we found that due to the difficulty of accessing live brain tissue, studies on ncRNA alterations in anxiety disorders rely largely on postmortem analyses from patients with psychiatric comorbidities, such as major depressive disorder, bipolar disorder, and schizophrenia, which complicates the interpretation of anxiety-specific findings [17,32,55]. In addition to this, in the following paragraphs we will discuss contradictory results, clinical translational bottlenecks, and sex-biased lncRNAs in the brain.

### 6.1. Contradictory Results

Limitations must be acknowledged. The complexity of the interactions between neuroimmune and neuroendocrine signaling is not yet completely understood; the interconnection between these two systems is lacking full characterization [74]. Differing experimental models, even though they offer valuable insights, have inherent differences across species and lead to variations in the interpretation of results, as well as influencing their generalizability [74,90,127].

Inconsistencies among the uniformity-lacking clinical trials that studied the influence of immunity on psychiatric disorders make it hard to draw definitive conclusions, due to the variations in sample size, population characteristics, and diagnosis criteria as well as methodological approaches, being some of the key aspects that add challenges in result interpretation and complicate the standardization of trials [74,127,128,129].

The heterogeneity of the results of studies conducted in psychiatry to understand the role of cytokines represents a challenge to interpret [129]. It is also important to address that it is not completely understood how peripheral blood cytokine and lncRNA levels reflect the actual ongoing situation in the brain [128].

Another important aspect to mention is the stage of a disorder; lncRNAs and cytokines have timely mechanisms of action, and their role varies through disease onset, symptom exacerbations, and evolution. The elevation of pro-inflammatory cytokines depends on the acute or chronic stress response, IL-1β was found to be increased by 38.5% in acute stress and 75.6% in chronic stress [75]. It is worth mentioning that comorbid diseases also have an impact on altered brain functions [128].

It is important to acknowledge the pleiotropic, redundant, synergistic, and antagonistic effects that cytokines [128]. IL-6 is an interesting cytokine as it is one of the most studied and has shown contradictory results [27]. It has been proven to have pro- and anti-inflammatory properties, depending on the presence of either an IL-6 receptor (pro-inflammatory) or a membrane-bound glycoprotein 130 transducer (anti-inflammatory) [127]. Moreover, studies have found no correlation between peripheral levels of IL-6 and cerebrospinal fluid levels, which suggests that peripheral levels do not reflect central IL-6 levels directly [127]. Additionally, the MALAT1 regulation of IL-6 has had some contradictory results as well, with overexpression associated with the downregulation of IL-6 (trauma brain injury inflammation) and overexpression associated with elevated IL-6 levels (acute myocardial infarction) [90,106].

### 6.2. Clinical Translational Bottlenecks

The potential of RNA-based therapies, such as anti-microRNAs and microRNA mimics, to restore normal gene expression is currently being actively studied [130]. However, even with miRNAs, challenges still arise, such as with their commercialization, since many present problems with medical administration and regulatory approval. Another challenge is that the regulatory pathways for microRNAs have not yet been fully developed, and their safety as well as efficacy must be thoroughly evaluated. In this way, microRNA-based therapies have the potential to revolutionize treatments for gene regulation and, therefore, influence the future of RNA therapeutics [131].

We believe that something similar is happening with lncRNAs, since, as already mentioned, several lncRNAs in various diseases have been found to be very promising biomarkers for diagnosis, prognosis, and responses to treatment. However, many lncRNA studies have a limited sample size and are limited to determining their biological function, which represents an obstacle to their inclusion as molecular biomarkers of clinical use [132,133].

Regarding the technical aspects, it must be acknowledged that, although the techniques for detecting lncRNAs have evolved and are quite sensitive, when quantifying lncRNAs it is important to select high-quality RNA, so the selection of the RNA extraction method is especially important, especially for complex samples such as plasma or serum, since it significantly influences the quality of the results and the robustness of the data [134].

Regarding the characteristics of lncRNAs, it is worth mentioning that some authors mention that, in general, the search for lncRNAs prioritizes the search for variants with higher expression levels, since transcripts with weak expression are usually overlooked; however, recent research suggests that lncRNAs expressed at low levels could be crucial for the function of the lncRNA; therefore, it is advisable to analyze transcripts at all expression levels [135].

As long as research keeps contributing to better understanding the role of lncRNAs and helps elucidate how their mechanisms work in dynamic assemblies with other macromolecules, the previous challenges will be resolved [20].

### 6.3. Sex-Biased LncRNAs in the Brain

Sex bias is present in all animals and manifests particular features across species and lineages [136]. LncRNAs are no exception, in fact, they regulate sex-biased protein-coding genes lineage-specifically; unfortunately, the impact of lncRNAs in this matter remains poorly understood [137]. Rodríguez-Montes et al. found that microglia, astrocytes, and oligodendrocytes tend to have more variety in gene expression across species compared to neurons [138]. He et al., based on RNA-seq datasets from human and macaque brain regions, found that, in humans, most sex-biased lncRNA target genes are enriched for immune-related functions [136].

Many psychiatric disorders are characterized by a strong sexual difference [3]. ADs are almost two times more likely to be developed by females, which points out the relevance of understanding the basis of sex differences in mental disorders, as it might provide key insights and open opportunities to search for sex-specific treatments [3,139].

The lncRNA XIST, master regulator of X-chromosome inactivation in females, has been proposed to function as competitive endogenous RNA (ceRNA) in a network where different types of ncRNAs can regulate each other’s expressions and compete by binding to other RNA targets [137]. In the literature, interaction between the XIST and miRNAs has been described, functioning as a sponge of different miRNAs to repress mRNAs [137,140]. However, the impact of ceRNA networks concerning sex-specific lncRNAs, their function, and mechanisms, is poorly studied [137]. It is worth noting that up to 15% of genes in the X chromosome escape inactivation, and the number and tissue distribution within an individual, but also between individuals, are largely variable [137].

Even though the molecular mechanisms linking sex-biased pathological basis are understudied, four models have been proposed to explain this phenomenon: (1) the major influence of hormonal levels between males and females, (2) gene expression carries different relevance in both sexes, (3) specific susceptibility factors encoded on the sexual chromosomes (X or Y), and the (4) multifactorial liability threshold between sexes [141,142,143].

## 7. Conclusions

LncRNAs in psychiatric disorders offer many challenges and opportunities for investigation; there are many questions with missing answers in the understanding of their functional implications in AD physiopathology. LncRNAs appear to be highly specific between species, which raises large challenges along the way of studying them, but it also makes them unique targets in the biomedical field [59].

MALAT1 has shown neuroplasticity-modifying mechanisms and is abundantly expressed by neurons [47]. This lncRNA responds to neuron activation, enhancing the transcription of genes related to stimuli-processing neuroplasticity, which evidence highlights as an important process in fear processing and adaptative behaviors that reduce anxiety symptoms [47,59].

GOMAFU is an interesting example of a brain-relevant lncRNA, with the potential to regulate anxiety behaviors; neural activation induces the downregulation of this lncRNA, in vivo and in vitro [54,144]. It has been proposed that GOMAFU negatively regulates genes involved in sustaining anxiety, like the Crybb1 gene [59,65]. In anxiety-inducing trials on mouse models, such as fear conditioning, GOMAFU undergoes a transient downregulation to allow the expression of genes that promote adaptative fear and vigilance [55,59,111]. This lncRNA looks promising as a biomarker and therapeutic target, considering its stress-dependent regulation to favor transcription of pro-anxiety genes [59].

NEAT1 is another remarkable lncRNA, as a brain-excitability-modifying molecule [51,59]. NEAT1 is a critical scaffold component and structural determinant of paraspeckles; it is involved in several cellular functions, including transcriptional regulation through chromatin structure modifications and splicing [145]. NEAT1, like GOMAFU, shows sensitive expression to neuronal activation [59]. The downregulation of this lncRNA increases excitability levels in the brain via glutamatergic activity, an important feature in AD pathogenesis [59].

LncRNAs are key regulators of neurodevelopmental processes; neuromodulation and brain signaling are important for proper nervous system function. Their dysregulation has been associated with psychiatric, neurodegenerative, metabolic, and other chronic disorders [63]. LncRNAs influence gene expression, synaptic plasticity, and neuroimmune responses to aid in establishing the active equilibrium required for emotional and cognitive homeostasis.

Chronic-stress-driven neuroinflammation exacerbates brain dysregulation, contributing to anxiety-related behaviors. The interplay between lncRNAs’ epigenetic mechanisms in brain functioning have a deeply complex network of interactions with the neuroendocrine system, the modulation of neural pathways, and neuroinflammation; further research is needed to elucidate key mechanisms to complete this puzzle.

The emerging evidence suggests that lncRNA dysregulation may play a role in AD onset and progression, positioning it as a potential biomarker and therapeutic target. Understanding lncRNA mechanisms may provide valuable insights into developing novel strategies with which to diagnose and treat anxiety disorders in a more precise and personalized way.

## Figures and Tables

**Table 1:** LncRNA regulatory mechanisms in neurodevelopmental processes and their connection to anxiety.

lncRNA	Regulatory Mechanism	Anxiety Connection	References
BDNF-AS	Represses the BDNF gene by recruiting PRC2; affects dendrite spine growth, neurogenesis.	Changes in spine density and neurogenesis contribute to anxiety-related behaviors in mouse models.	[43,44]
MALAT1	Regulates synaptogenesis and neuroplasticity by modulating gene expression.	MALAT1 downregulation has been linked to reduced synaptic density and neuron apoptosis in the hippocampus in mouse models, which has been found to reinforce hyperactive fear responses.	[21,43,48]
NEAT1	Maintains paraspeckle integrity. Regulates alternative splicing. Prevents neuron apoptosis.	NEAT1 downregulation alters alternative splicing of genes important to enabling adaptability in stress responses in mouse models.	[21,22,51]
GOMAFU	Binds DISC1, ERBB4, and WNT7B; regulates alternative splicing patterns, neurogenesis, glial cell differentiation, neuroplasticity, and neuron survival.	The downregulation of GOMAFU can alter brain excitability, and dysregulation is also associated with neuroinflammation, which promotes anxiety-like behaviors in mouse models.	[43,53,55,56]
Evf2	Recruits DLX family genes and MECP2; controls the differentiation of GABAergic neurons in the hippocampus and dentate gyrus of mice.	GABAergic neurons play an important role in counterbalancing brain hyperactivity during and after stress responses and sustained excitatory states, especially on limbic system regions, contributing to anxiety-like behaviors.	[42,43]
PNKY	Interacts with PTBP1; regulates the expression and alternative splicing of gene transcripts that promote neurogenesis and migration in embryonic NSCs.	Studies in mice show that neurogenesis impairment enhances anxiety-like behaviors, especially in the hippocampus.	[42,43,46]
BC1/BC200	Regulates local protein synthesis in synapses; modulates neuronal excitability and plasticity.	Studies in mouse models have shown that the absence of BC1/BC200 leads to altered glutamatergic transmission and maladaptive anxiety behaviors.	[21,43,57]

**Table 2:** Mechanisms of lncRNAs associated with neuroinflammation.

LncRNA	Mechanism(s)	References
BDNF-AS	Inhibits BDNF expression by recruiting repressive histone marks to its promoter. BDNF-AS upregulation promotes neurotoxicity as well as apoptosis and decreases cell viability.	[22,73,88]
GOMAFU	Regulates brain transmission through dopamine and glutamate pathways. Negatively modulates the IFN-γ pathway.	[55,64]
MALAT1	Controls gene expression linked to synaptogenesis through SR protein interactions. Acts as a sponge for miR-125b, inhibiting neuron apoptosis and inflammation. Highly expressed in neurons. Dysregulation is associated with neuroinflammation.	[21,22,48]
NEAT1	Acts as a sponge for miR-212-5p. Involved in paraspeckle body formation and regulates microglial activation. Part of the unfolded proteins response in cellular stress. Upregulates the expression of NLRP3 in macrophages, promoting occurrence of inflammatory responses. NEAT1 downregulation has been shown to reduce levels of IL-1β and TNF-α.	[21,88,90,91]
MEG3	Acts as a miRNA sponge. Inactivates the PI3/AKT signaling pathway. Downregulates the NF-κB signaling pathway. Targets the miR-7a-5p/NLRP3 axis to regulate microglia activation and inflammatory response.	[22,88,90]
TUG1	Acts as a sponge for miR-9 and miR-145a-5p, activating the NF-κB signaling pathway.	[22,88,92]
H19	Promotes microglial and astrocyte activation by activating the JAK/STAT pathway.	[88,93]
Gm4419	Serves as a decoy by binding and phosphorylating IkBα.	[88,94]
NKILA	Inhibits the NF-κB signaling pathway.	[88]
RMST	Activates the NF-κB signaling pathway. Favors microglial activation and neuronal apoptosis.	[88,95]
HOTAIR	Histone methylation and acetylation, functions as a scaffold for chromatin-remodeling complex PRC2. Glial activation in neuroinflammatory responses.	[22,96]
LncRNA Nespas	Inactivates the NF-κB signaling pathway via the suppression of TAK1.	[97]
GAS5	Binds to PRC2, inhibiting M2 polarization. Acts as a sponge for miR-223-3p and positively regulates the NLRP3 inflammasome.	[88]
LincRNA-p21	Competitively binds to the miR-181 family, inducing microglial activation.	[88]
LincRNA-Cox2	Directly binds and promotes the NF-κB-p65 nuclear translocation and transcription of NLRP3.	[88]

**Table 3:** Examples of lncRNA as possible biomarkers in different diseases.

Disease	Trial	LncRNA(s)	Sample	Reference
Acute myeloid leukemia (AML)	137 patients 43 controls	↑ FBXL19 antisense RNA 1 (FBXL19-AS1) in AML and overexpression associated with a bad prognosis.	Serum	[104]
Acute myeloid leukemia (AML)	119 patients 26 controls	↑ Promoter of CDKN1A antisense DNA damage-activated RNA (PANDAR) in AML, and a higher expression is associated with poor clinical outcomes.	Bone marrow	[105]
Acute myocardial infarction (AMI)	160 patients (newly diagnosed with AMI) 50 controls (angina pectoris patients, no AMI)	↑ MALAT1 in AMI and was positively correlated with CRP, troponin I, LDL, and infarct size, as well as TNF-alpha, IL-6 and IL-17A.	Peripheral blood	[106]
Type 2 diabetes (T2D)	100 patients 100 controls	↑ lncRNA XR_108954.2 and E2F2 mRNA in T2D	Peripheral blood	[107]
COVID-19	38 moderate and 25 severe COVID-19 patients 30 controls	↑ ANRIL, THRIL and NEAT1 in COVID-19 patients. ANRIL and THRIL higher in severe vs. moderate. NEAT1 higher in both (moderate and severe) without significant difference.	Peripheral blood	[108]
Bipolar disorder (BD)	130 patients 116 controls	↑ lncRNA NR_028138.1.	Peripheral blood	[109]
Schizophrenia (SZ)	106 patients 48 controls	↑ NONHSAT089447 and NONHSAT041499 in SZ; both showed a significant reduction after treatment.	Peripheral blood	[110]
Schizophrenia (SZ)	35 patients 49 controls	↑ GOMAFU in SZ.	Peripheral blood	[111]

↑: upregulated/overexpressed.

**Table 4:** Expression patterns of lncRNAs and miRNAs in anxiety-related disorders.

Disorder	Trial	LncRNA(s)/MiRNA(s)	Sample	Reference
Post-traumatic stress disorder	30 patients 42 controls	↑ miR-570, miR-219, miR-637, miR-668, miR-519a, miR-518f, and miR-615. ↓ miR-125a and miR-181c.	Peripheral blood	[116]
Generalized anxiety disorder	76 patients 39 controls	↑ miR-4484, miR-4674, miR-501, miR-663, and miR-4505. ↓ miR-1301 and miR-432.	Peripheral blood	[117]
Social stress	49 healthy	↑ miR-29c.	Peripheral blood	[118]
Anticipatory anxiety	10 healthy medical students	↑ miR-144 and miR-16, associated with an upcoming stressful exam.	Blood plasma	[119]
Anxiety proneness	88 patients (adolescents with childhood trauma)	↑ hsa-miR-28-5p. ↓ hsa-miR-502-3p and hsa-miR-500a-3p.	Peripheral blood	[120]
Depression/anxiety	181 patients 59 controls	↑ Mitochondiral 7S RNA.	Blood plasma	[72]

↑: upregulated/increased; ↓: downregulated/decreased.

## Data Availability

No new data were created or analyzed in this study. Data sharing is not applicable to this article.

## Abbreviations

The following abbreviations are used in this manuscript:

ADs	Anxiety disorders
AKT	Protein kinase B
AMI	Acute myocardial infarction
AML	Acute myeloid leukemia
ASOs	Antisense oligonucleotides
B200	Brain cytoplasmic 200 lncRNA
BC1	Brain cytoplasmic 200 lncRNA
BD	Bipolar disorder
BDNF	Brain-derived neurotrophic factor
BDNF-AS	Brain-derived neurotrophic factor antisense RNA
ceRNA	Competitive endogenous RNAs
circRNAs	Circular RNAs
CNS	Central nervous system
CRH	Corticotropin-releasing hormone
CRHBP	CRH binding protein
DISC1	Disrupted in schizophrenia 1
DLX	Distal-less homeobox
ERBB4	Erb-B2 receptor tyrosine kinase 4
ESR1	Estrogen receptor 1
Evf2	Embryonic ventral forebrain 2
GABA	Gamma-aminobutyric acid
GAD	Generalized anxiety disorder
H19	H19 maternally imprinted expressed transcript
HOTAIR	HOX transcript antisense RNA
HPA	Hypothalamic–pituitary–adrenal
IL	Interleukin
JAK/STAT	Janus kinase/signal transducers and activators of transcription
JNK	c-Jun N-terminal kinases
lincRNA-p21	Long intergenic ncRNA p21
lncRNA(s)	Long non-coding RNA (s)
lncRNA-COX-2	Cyclooxygenase 2 lncRNA
MAD1L1	Mitotic arrest deficient 1 like 1
MALAT1	Metastasis-associated lung adenocarcinoma transcript 1
MECP2	Methyl-CpG binding protein 2
MEG3	Maternally expressed gene 3
MIAT	Myocardial infarction-associated transcript
miRNA(s)	MicroRNA (s)
MKK4	Mitogen-activated protein kinase kinase 4
mRNA	Messenger RNA
NATs	Natural antisense transcript (s)
ncRNA (s)	Non-coding RNA (s)
NEAT1	Nuclear-enriched abundant transcript 1
NF-κB	Nuclear factor kappa B
NKILA	NF-KappaB interacting lncRNA
NRLP3	NLR family pyrin domain containing 3
NSC	Neural stem cell
PI3K	Phosphoinositide-3 kinases
PRC2	Polycomb repressive complex 2
PTBP1	Polypyrimidine tract binding protein 1
PTSD	Post-traumatic stress disorder
RMRP	Mitochondrial RNA-processing endoribonuclease
RMST	Rhabdomyosarcoma 2-associated transcript
siRNAs	Small interfering RNAs
SNP	Single-nucleotide polymorphism
SNPs	Single-nucleotide polymorphisms
sRNA	Small RNA
STAB1	Stabilin 1 protein coding gene
SZ	Schizophrenia
T2D	Type 2 diabetes
TAK1	Transforming growth factor-β-activated kinase 1
TLR	Toll-like receptor
TNF-α	Tumor necrosis factor alpha
TUG1	Taurine up-regulated 1
WNT7B	Wingless-type family member 7B
